# Microseismic P-Wave Travel Time Computation and 3D Localization Based on a 3D High-Order Fast Marching Method

**DOI:** 10.3390/s21175815

**Published:** 2021-08-29

**Authors:** Yijia Li, Jing Wang, Zhengfang Wang, Qingmei Sui, Ziming Xiong

**Affiliations:** 1 School of Control Science and Engineering, Shandong University, Jinan 250061, China; liyijia@mail.sdu.edu.cn (Y.L.); wangjingkz@sdu.edu.cn (J.W.); qmsui@sdu.edu.cn (Q.S.); 2 State Key Laboratory for Disaster Prevention and Mitigation of Explosion and Impact, Army Engineering University of PLA, Nanjing 210007, China; xzm992311@163.com

**Keywords:** microseismic monitoring, three-dimensional high-order fast marching method, P-wave travel time computation, 3D localization of microseismic sources

## Abstract

The travel time computation of microseismic waves in different directions (particularly, the diagonal direction) in three-dimensional space has been found to be inaccurate, which seriously affects the localization accuracy of three-dimensional microseismic sources. In order to solve this problem, this research study developed a method of calculating the P-wave travel time based on a 3D high-order fast marching method (3D_H_FMM). This study focused on designing a high-order finite-difference operator in order to realize the accurate calculation of the P-wave travel time in three-dimensional space. The method was validated using homogeneous velocity models and inhomogeneous layered media velocity models of different scales. The results showed that the overall mean absolute error (MAE) of the two homogenous models using 3D_H_FMM had been reduced by 88.335%, and 90.593% compared with the traditional 3D_FMM. On that basis, the three-dimensional localization of microseismic sources was carried out using a particle swarm optimization algorithm. The developed 3D_H_FMM was used to calculate the travel time, then to conduct the localization of the microseismic source in inhomogeneous models. The mean error of the localization results of the different positions in the three-dimensional space was determined to be 1.901 m, and the localization accuracy was found to be superior to that of the traditional 3D_FMM method (mean absolute localization error: 3.447 m) with the small-scaled inhomogeneous model.

## 1. Introduction

Microseismic monitoring technology is a geophysical technology used to evaluate the impacts, effects, and underground state of production activities through the observations and analyses of microseismic events [1]. This type of technology has been widely used in mining [2,3], tunnel construction [4,5], slope [6,7], hydraulic fracturing [8,9], reservoirs [10,11], and other fields. Accurate microseismic localization methods are the basis of stability analysis and the study of microseismic monitoring technology. The accurate computation of P-wave travel time is considered to be one of the important factors when determining the accuracy of microseismic localization. For example, if the calculation of the P-wave travel time is inaccurate, it will have a major impact on the microseismic source localization results. Therefore, due to the significance of accurate computations of first phase travel time, Chinese and international researchers have carried out detailed investigations from the perspective of ray-tracing [12,13,14,15,16,17]. For example, improved ray-tracing methods have been used to calculate the travel time of gently inclined strata [12]. The finite-differences (FD) of rotationally staggered grids have been utilized to calculate travel times [13]. Fast marching methods (FMM) with local grid refinement (LGRs) combined with embedded discrete fracture models (EDFM) have been successful in simulating high-frequency fractures [14]. In addition, FMM methods have been adopted to solve the arrival time values of the Eikonal equation in spherical coordinates [15]. Other research investigations have applied multi-template fast marching methods (MSFM) to calculate the travel times of microseismic waves [16]. In addition, improved algorithms combining FMM with linear interpolation ray-tracing methods (LTI) have also been successful in calculating the travel times of microseismic waves [17]. Rawlinson et al. studied the propagation of seismic waves in a two-dimensional layered medium, using the FMM method to track multiple reflection and transmission phases in the medium [18]. This study found that the majority of the aforementioned methods have focused on calculating the P-wave travel time and locating microseismic sources in two-dimensional space. However, in practical engineering, it is often necessary to locate microseismic sources in three-dimensional space.

In recent years, microseismic localization in three-dimensional space has become a research hotspot and future development trend [19,20,21,22,23,24,25,26]. In the field of Wireless Sensor Network (WSN), Peng and Wang et al. proposed an optimized novel terrain deployment scheme to improve the coverage of the network, which was applied to the localization problem on 3-D terrain [19,20]. While in terms of time difference accuracy, event properties, initial velocity models, and back-propagation techniques, 3D localization of microseismic sources has also been rapidly developed [21,22,23,24,25,26]. Wang et al. developed a semiautomatic waveform cut method based on the cross-correlation to determine the direct P-phase relative delay times [21]. Tang et al. measured the fracture geometry and orientation for 3D discrete fracture networks modeling based on the domain classification methodology [22]. Dong et al. developed a velocity-free MS/AE source method to achieve localization of 3D pore-containing structures [23]. Zhang et al. performed a constant velocity localization exercise to extract a preliminary and more accurate velocity model [24]. Peng et al. presented a 3D heterogeneous velocity model for excavated tunnels [25]. Wang et al. used a high-resolution tomographic 3D velocity model for wavefield back-propagation [26]. Among the available arrival time acquisition methods, the method of wavefront expansion based on finite-difference grid element has become the mainstream method of the travel time computation of microseismic waves and has been gradually applied in three-dimensional space [27,28,29,30,31,32,33,34,35]. Vidale et al. were the first to extend the time values obtained by finite-difference methods to three-dimensional cases [27]. Guo et al. established a three-dimensional fast sweeping method (FSM) in a Cartesian coordinate system for the purpose of calculating the P-wave travel time [28]. Sethian et al., Hassoun et al., and Popovici et al. derived a complex 3D FMM method in order to calculate the travel time processes [29,30]. Jiang et al. calculated the theoretical arrival time of microseismic waves in a cavern-containing model [31]. Kumbhakar et al. applied a 3D FMM method to solve the travel time responses of various underground numerical models [32]. In another related study, Lou applied 3D FMM to three-dimensional transversely isotropic media (TTI) with an inclined symmetry axis for the purpose of calculating the travel times of microseismic waves [33]. Peng et al. utilized the traditional 3D FMM to calculate the arrival time of the 3D velocity model [34]. According to the different media of velocity models, the above-mentioned methods improved the extraction accuracy of travel times in a specific direction of microseismic three-dimensional space, and obtained relatively accurate travel times for microseismic waves. However, first-order operators were mostly used for the approximate solutions in the above-mentioned methods, and the accuracy of the travel times of the microseismic waves calculated in all directions of three-dimensional space were found to be inconsistent. For example, the errors were observed to be the greatest along the 45° diagonal of the grid [14,16,33,34], which led to the inaccurate calculation of the travel times of the entire velocity model. This had resulted in a large number of microseismic source localization errors. Therefore, it is urgent to identify a method for accurate computation of the P-wave travel time. In this study, the researchers believed that focus should be placed on solving the problem of large diagonal direction errors in order to improve the accuracy of the P-wave travel time selection within an entire 3D space.

Therefore, in order to solve the above-mentioned accuracy problems, a 3D high-order fast marching method (3D_H_FMM) was developed, which focused on the introduction of high-order operators and realized more accurate computation of the travel times and localization of microseismic sources. First of all, a 3D_H_FMM was established in a Cartesian coordinate system. Its validity and accuracy were verified using homogeneous velocity models. The results showed that the overall mean absolute error (MAE) of the two homogenous models using 3D_H_FMM had been reduced by 88.335% with the small-scaled homogeneous model, and 90.593% with the large-scaled homogeneous model, compared with the traditional 3D_FMM. The developed method improved the extraction accuracy of the full space travel time, which fully proved the validity and veracity of the developed method.

Then, the developed method was applied to inhomogeneous layered velocity models, and a particle swarm optimization algorithm was used to locate the microseismic sources in three-dimensional space. The mean error of the localization results of the different positions in the three-dimensional space was determined to be 1.901 m, and the localization accuracy was found to be superior to that of the traditional 3D_FMM method (mean absolute localization error: 3.447 m) with the small-scaled inhomogeneous model, while the mean error of the localization results of 3D_H_FMM was 42.764 m (compared to that of 3D_FMM: 62.092 m) with the large-scaled inhomogeneous model. The source localization accuracy had been significantly improved, which further verified the superiority of the developed method. The results obtained in this study provided certain technical support for calculating the travel times and source localization in microseismic monitoring processes, and the developed method was considered to have good engineering application prospects.

## 2. P-Wave Travel Time Calculating Method

**Figure 1:**
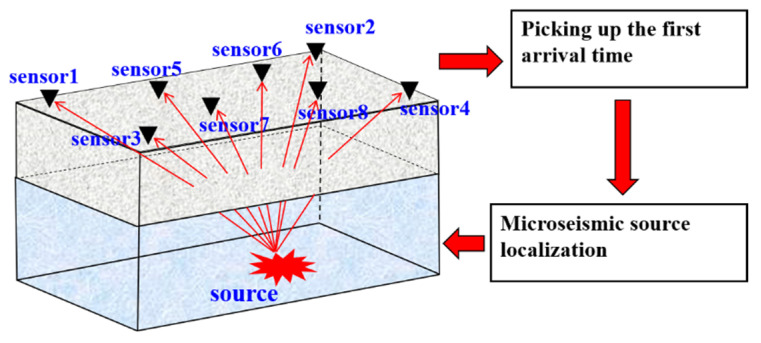
Schematic diagram of the microseismic monitoring system.

Figure 1 shows a typical microseismic monitoring system in a distributed sensor network. The sensors are arranged above the geological velocity model, and microseismic sources are distributed below the geological velocity model. The travel time is calculated by the method of wavefront expansion with finite-difference grid element and then used to realize the microseismic source localization.

In view of the inconsistencies in the accuracy of P-wave travel times in three-dimensional space, and the large number of errors encountered in determining the travel time computations in the direction of 45°, this study developed a calculating method of P-wave travel time based on a 3D high-order fast marching method. The developed method was designed with a high-order finite-difference operator to replace the traditional first-order operator, and a local calculation method was adopted to derive the travel times of the velocity model nodes. This reduced the errors of P-wave travel time in the diagonal direction of the velocity model, and improved the accuracy of the travel times of the entire three-dimensional space velocity model. The following section briefly introduces the working principle of traditional FMM in two-dimensional space and the process of wavefront expansion. On that basis, it then focuses on the introduction of three-dimensional high-order FMM.

### 2.1. Fast Marching Method

The propagation of microseismic waves in underground media can be described by various wave equations in elastodynamics. Under the assumptions of high-frequency approximations (for example, the signal wavelengths will be much smaller than the inhomogeneity of microseismic wave propagation medium), the elastic wave equation for a p-wave in an isotropic medium can be expressed as follows:

(1) $$\nabla^{2}\phi -\frac{1}{\alpha }\frac{\partial^{2}\phi }{\partial^{2}t}=0$$

where ϕ represents the scalar potential function of the P-wave, α represents the wave velocity of the P-wave, and t is the time. Then, using the high-frequency hypothesis (ω→∞), the equation can be obtained as follows:

(2) |∇T|=s

where s=1/v indicates slowness, and T(x) represents the travel time of an elastic wave. Therefore, when T is fixed, it represents an equipotential surface (for example, a wavefront). According to the physical meaning of the Eikonal equation, it can be assumed that the P-wave travel time at different positions can be obtained by solving the Eikonal equation.

FMM is a type of finite-difference numerical method based on network expansion. Monitoring areas are meshed when using this type of method, and an upwind difference scheme is used to solve the equation at each node. Among those, narrow band expansions and heap sorting are two key technologies used in FMM in order to calculate travel times. In addition, narrow band technology is used to simulate the wavefront propagation process, and heap sorting techniques are used to determine the minimum travel time in microseismic velocity models [14,15,18].

### 2.2. 3D High-Order Fast Marching Method

In order to solve the problem of the low extraction accuracy of the travel times in the diagonal direction of three-dimensional space, a three-dimensional high-order fast marching method was adopted for P-wave travel time extraction process of microseismic waves [29,30]. This study focuses on the development of a higher-order difference operator and its application to a microseismic 3D laminar velocity model to calculate node travel times.

In the Cartesian coordinate system, the Eikonal equation corresponding to the wave equation can be expressed as follows:

(3) $$\sqrt{{\left(\frac{\partial t}{\partial x}\right)}^{2}+{\left(\frac{\partial t}{\partial y}\right)}^{2}+{\left(\frac{\partial t}{\partial z}\right)}^{2}}=s(x,y,z)$$

The three-dimensional model was networked, and it was assumed that every node could be obtained. An upwind difference operator was used to discretize the model, and the discrete function equation was obtained as follows:

(4) $${\left|\nabla t\right|}_{i,j,k}={\left[\begin{array}{c} \max(D_{n}{}^{-x}t_{i,j,k},D_{n}{}^{+x}t_{i,j,k},0)\\ \max(D_{n}{}^{-y}t_{i,j,k},D_{n}{}^{+y}t_{i,j,k},0)\\ \max(D_{n}{}^{-z}t_{i,j,k},D_{n}{}^{+z}t_{i,j,k},0) \end{array}\right]}^{1/2}=s_{i,j,k}$$

where (i,j,k) represents the incremental variables in the three-dimensional Cartesian grid (x,y,z), and an upwind finite-difference operator was used in Formula (4); $D_{n}^{-x}t_{i,j,k}$ indicates the backward difference in the x direction; $D_{n}^{+x}t_{i,j,k}$ denotes the forward difference in the x direction, and the other four operators have the same form. The integer variable *n* = 1/2/3 represents the order of the finite-difference operator of the 3D fast marching method with a default value of *n* = 1, which is referred to as a 3D fast marching method (3D_FMM); and when high-order operators are used, it is referred to as a 3D high-order fast marching method (3D_H_FMM).

If the calculation results of a high-order operator are not acceptable in the process of using 3D_H_FMM to calculate travel time, a lower order operator is often used to replace it. Strictly speaking, the second-order and third-order algorithms belong to hybrid algorithms. The accuracy and computational efficiency of first-order, second-order, and third-order operators were compared and analyzed, as shown in References [29,31,34]. The results showed that the accuracy of first-order FMM was too low. There was little difference observed between the accuracy levels of the second-order and third-order, but the computational time of the third-order was much longer than that of the second-order. Therefore, second-order operators are often used for FMM calculations. In this study, the higher order refers to the use of a second-order operator for the finite-difference in order to calculate the P-wave travel time values. If $t_{i-1,j,k}<t_{i-2,j,k}$, the first-order operator is used for calculation. Then, $D_{n}^{-x}t_{i,j,k}$ in Equation (4) can be expressed as:

(5) $$\{\begin{array}{ll} D_{1}^{-x}t_{i,j,k}=\frac{t_{i,j,k}-t_{i-1,j,k}}{△x} & \begin{array}{cc} t_{i-1,j,k} & \mathrm{exist}; \end{array}\\ D_{1}^{+x}t_{i,j,k}=\frac{t_{i+1,j,k}-t_{i,j,k}}{△x} & \begin{array}{cc} t_{i+1,j,k} & \mathrm{exist}; \end{array}\\ D_{2}^{-x}t_{i,j,k}=\frac{3t_{i,j,k}-4t_{i-1,j,k}+t_{i-2,j,k}}{2△x} & \begin{array}{cccc} t_{i-1,j,k},t_{i-2,j,k} & \mathrm{exist} & \& & t_{i-1,j,k}>t_{i-2,j,k} \end{array}\\ D_{2}^{+x}t_{i,j,k}=\frac{3t_{i,j,k}-4t_{i+1,j,k}+t_{i+2,j,k}}{2△x} & \begin{array}{cccc} t_{i+1,j,k},t_{i+2,j,k} & \mathrm{exist} & \& & t_{i+1,j,k}>t_{i+2,j,k} \end{array} \end{array}$$

**Figure 2:**
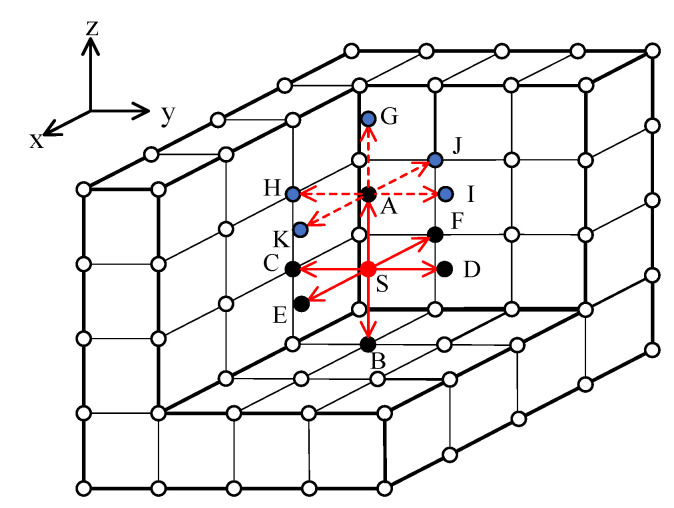
Three-dimensional FMM calculation example.

Taking a cube calculation model as an example, it was assumed that the number of grid nodes in the model was n×n×n. Figure 2 describes the detailed process of solving the three-dimensional coordinate function equation. The two-step expansion process is included in the figure, and the specific implementation steps were as follows:

(1) Initialization node: First, the initial state was that the travel time at source point S was 0. Second, the travel times of six nodes around the source point were calculated to form the initial narrow band point, while all the other grid nodes were the unfinished points.

(2) Narrow band extension: A 3D_H_FMM was used to calculate travel time, and Points A, B, C, D, E, and F were nodes with known travel times. Therefore, if the minimum travel time point from the known nodes was chosen, it could be assumed that Point A was the current extension point. Then, if Point A was the receiving point, and Points G, H, I, J, and K were the points to be calculated, they form a new narrow band together with B, C, D, E, and F. The node with the smallest travel time in the narrow band was then selected for expansion. Then, the narrow band was expanded and the above steps were repeated until there were no nodes left in the narrow band. At that point, the calculation process was ceased. During the previously described process, at a certain time and a certain point, the travel times of multiple nodes (A, B, C, D, E, F) in six directions became known, and the local calculations of the travel times of the nodes could be realized using Formula (4).

In the process of calculating P-wave travel time, the ray paths from the simulated source points to all sensors directly, and when encountering layered interfaces with different velocities, satisfy Snell’s law [12,13,14,15,16,17,18], as shown in Figure 1. The specific expression formula is, $\sin\theta_{1}/\sin\theta_{2}=v_{1}/v_{2}$ where $v_{1}$, $v_{2}$ are two adjacent wave velocity values, $\theta_{1}$ is the angle of incidence, $\theta_{2}$ is the angle of refraction [34].

## 3. Validation and Analysis of the Travel Time Computation

Finite-difference-based solutions of functional equations have become the mainstream method for determining the P-wave travel time. A large number of the currently available studies have generally used simulation data for verification purposes [23,27,31,35,36,37,38]. Based on that, this study also utilized simulation data to verify the performance of the developed method. Velocity models of different media (including 3D homogeneous velocity models and inhomogeneous layered velocity models) were used to carry out forward calculations, and the travel times calculated by the developed method were verified and analyzed. The main phase identified in the process of microseismic elastic wave simulation is the transmission and refraction phase, which propagates directly from a microseismic source to the geophone. The first phase of travel time in this study refers to the P-wave travel time.

Differing from earthquakes, the frequencies of microseismic signals are high. However, the wavelengths of the signals are small, and the signal energy tends to become seriously attenuated during the propagation process. Small-scale and large-scale microseismic velocity models were adopted for experimental validation in this study. The specific scale ranges and sensor coordinates are shown in Table 1 below, respectively. Both cases have been validated in homogeneous and inhomogeneous models. Therefore, the cases used in this paper contain four kinds, namely, Case A: small-scaled homogeneous model, Case B: large-scaled homogeneous model, Case C: small-scaled inhomogeneous model, and Case D: large-scaled inhomogeneous model, with a grid of 1 m for small-scale models and 10 m for large-scale models.

**Figure 3:**
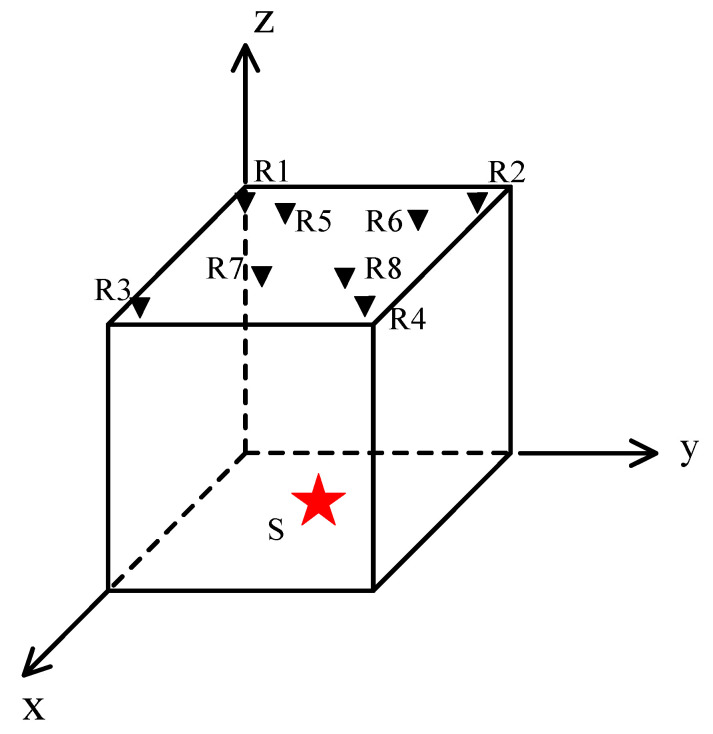
Schematic diagram of the microseismic source model.

A schematic diagram of the microseismic source model is shown in Figure 3. In the figure, the red five-pointed star S represents the source point, and the black inverted filling triangle point R indicates the receiving point. It contains 8 sensors, all of which are placed on the upper surface of the model, with 4 of them distributed in the corners of the model.

The distance from the source point to each node, divided by the velocity, was taken as the analytical solution. In other words, the theoretical value of arrival time of the first break, which was expressed as Equation (6). The calculation results of 3D_H_FMM and 3D_FMM were taken as the numerical solution. The difference between the absolute values of the numerical solution and the analytic solution was then taken as the absolute error at each node and expressed as Equation (7). The relative error value of the numerical and analytical solutions was taken as the error degree, and the accuracy was expressed as Equation (8). Therefore, according to the two methods, the MAE and the accuracy of the multiple nodes within the scope of the model were calculated, which were then expressed as Equations (9) and (10), respectively. The specific expressions are as follows:

(6) $$t_{A}{}^{i}=\sqrt{{\left(x_{i}-x\right)}^{2}+{\left(y_{i}-y\right)}^{2}+{\left(z_{i}-z\right)}^{2}}/v$$

(7) $$\delta t_{\mathrm{abs}}{}^{i}=\left|t_{N}{}^{i}-t_{A}{}^{i}\right|\;\;\;\;\left(N=1/2\right)$$

(8) $$\upsilon t_{acc,N}{}^{i}=1-\left(\left|t_{N}{}^{i}-t_{A}{}^{i}\right|/t_{A}{}^{i}\right)\;\;\;\;\left(N=1/2\right)$$

(9) $$\xi_{\mathrm{abs},N}=\frac{1}{n}\sum_{i=1}^{n}\left|t_{N}{}^{i}-t_{A}{}^{i}\right|\;\;\;\;\left(N=1/2\right)$$

(10) $$\tau_{acc,N}=\frac{1}{n}\sum_{i=1}^{n}\left[1-\left(\left|t_{N}{}^{i}-t_{A}{}^{i}\right|/t_{A}{}^{i}\right)\right]\;\;\;\;\left(N=1/2\right)$$

where $t_{A}{}^{i}$ represents the analytical solution at the node i; $t^{i}$ indicates the numerical solution at the node i; $\delta t_{\mathrm{abs}}{}^{i}$ denotes the absolute error at the node i; $\upsilon t_{acc}{}^{i}$ represents the accuracy at the node *i*; *ξ_abs_* indicates the mean absolute error (MAE) of multiple nodes; $\tau_{acc}$ refers to the accuracy of multiple nodes. The subscripts (N=1/2) correspond to 3D_FMM and 3D_H_FMM, respectively. The absolute error and accuracy results were used as the evaluation indexes for the P-wave travel time values, and the validity and accuracy of the developed method were verified through the P-wave travel time calculation and error analysis of the uniform model example. In this study, the smaller the absolute error was, the higher the accuracy of the results would be. We have summarized the main notations used in the paper, as shown in Table 2.

### 3.1. Homogeneous Velocity Model

First of all, Case A: small-scaled homogeneous model was adopted, and there was a total of $50^{3}=\;125,000$ nodes. The specific scale range and the positions of sensors are shown below in Table 1.

From the perspective of a three-dimensional homogeneous velocity model, the P-wave travel time calculated by the 3D_H_FMM was compared with that of the 3D_FMM and the theoretical value. Then, error analysis was carried out for the purpose of verifying the effectiveness and accuracy of the method presented in this study. In the three-dimensional homogeneous velocity model, the P-wave velocity (v) was a random value between 2000 m/s and 4000 m/s, and the source point S was randomly generated and irregularly distributed within the velocity model at a scale range of 50 m^3^.

**Figure 4:**
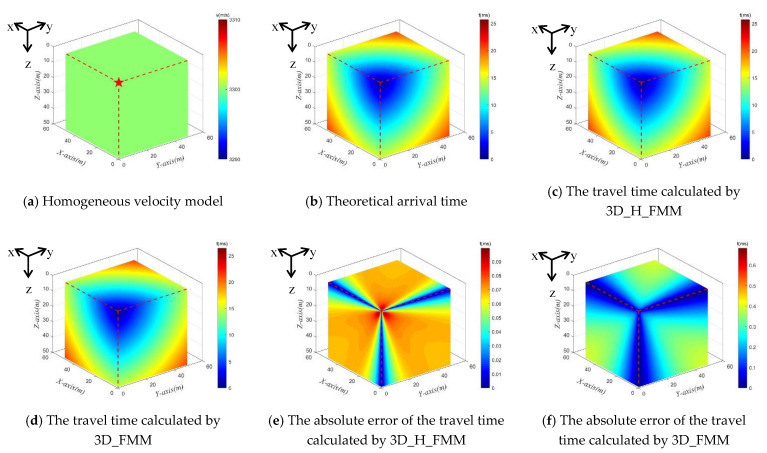
The Case A: small-scaled homogeneous model and its theoretical arrival time, the P-wave travel time and error distribution map calculated by 3D_H_FMM and 3D_FMM.

A single source in Cartesian coordinates was taken as an example, as shown in Figure 4a. The velocity of the P-wave was 3300 m/s, and the localization of the source point was the point (1, 1, 1). The theoretical value of P-wave travel time (for example, the analytical solution) was obtained using Equation (6), as detailed in Figure 4b. Both the 3D_H_FMM and 3D_FMM were used to calculate the numerical solutions of travel times, as shown in Figure 4c,d, respectively. The results revealed that the trajectory of travel times obtained using 3D_H_FMM and 3D_FMM were geometrically similar to the theoretical value, and the distribution shapes were basically the same. In the entire velocity model, the maximum theoretical value of the travel time (max.t) was 25.718 ms, and the maximum travel times calculated by the 3D_H_FMM and 3D_FMM were 25.773 ms and 26.403 ms, respectively. Those results confirmed the effectiveness of the method developed in this study.

The errors, namely the absolute errors, between the first phase travel time values determined by the 3D_H_FMM, 3D_ FMM, and its theoretical value were charted, as detailed in Figure 4e,f, respectively. As can be seen in the figures, in the entire velocity model, the absolute errors of the travel times of the 3D_H_FMM and 3D_FMM basically increased at the same rate in X, Y, and, Z directions, and the diagonal direction displayed a larger error distribution. The maximum absolute errors (max.err.) of the 3D_H_FMM and 3D_FMM were determined to be 0.100 ms and 0.685 ms, respectively. Therefore, based on the results, the maximum absolute errors calculated by the method developed in this study were far less than those of the 3D_FMM, at approximately 1/7, indicating that the developed method had achieved more accurate results.

**Figure 5:**
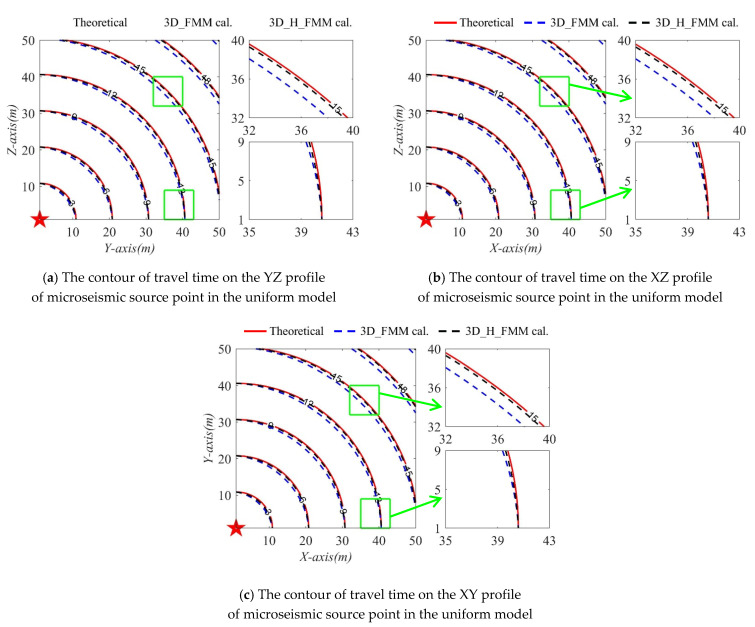
The contour of travel times of the three profiles of microseismic source point in the Case A: small-scaled homogeneous model.

In order to express the accuracy of the method developed in this study more clearly, the P-wave travel time values of the three profiles, x = 1 m (YZ profile), y = 1 m (XZ profile), and z = 1 m (XY profile), located at the source point, were retrieved. The contour lines of the travel times calculated by 3D_H_FMM and 3D_FMM, along with their theoretical values, are shown in Figure 5. Among those, small areas in the direction of the diagonal and the y-axis were amplified. By comparison, it could be seen that when compared with the 3D_FMM, the P-wave travel time calculated by the method developed in this study was closer to the theoretical value and the absolute error was smaller. In addition, when compared with the y-axis direction, the accuracy in the diagonal direction had been more obviously improved, as indicated in the green box plot in the figure. However, according to the contour distribution shown in Figure 5, the travel times of both the 3D_H_FMM and 3D_FMM were larger than their theoretical values at a specific node, with the P-wave travel time of the 3D_FMM being the maximum.

**Figure 6:**
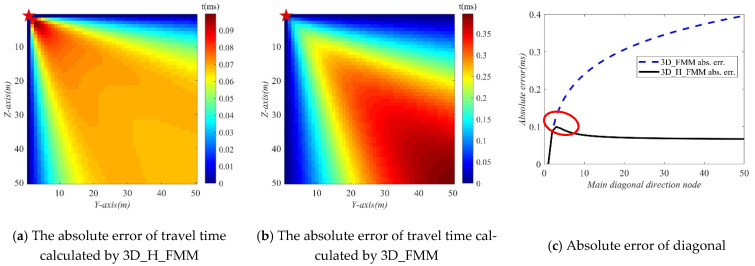
The absolute error, in the YZ profile, and its diagonal direction of the source point of the travel time calculated by 3D_H_FMM and 3D_FMM in the Case A: small-scaled homogeneous model.

In the present research investigation, in order to more intuitively reflect the accuracy of the presented method, the absolute error of the travel time of a single profile at the source point was graphed and analyzed, and then compared with that of the 3D_FMM. As detailed in Figure 5a, the YZ profile where the source point was located was taken as an example. The absolute errors of travel times calculated by the 3D_H_FMM and 3D_FMM are shown in Figure 6a,b, respectively. The absolute errors in the diagonal direction of that profile are shown in Figure 6c. As can be seen in the figure, in the YZ profile where the source point was located, the absolute error of travel time calculated by 3D_FMM was large in the diagonal direction and displayed an increasing trend, with a max.err. of 0.396 ms. The max.err. of the 3D_H_FMM was 0.100 ms, which was a relative reduction (MAE red. per.) of 74.856% ($\left(\delta t_{\mathrm{abs},2}{}^{i}-\delta t_{abs,1}{}^{i}\right)/\delta t_{abs,1}{}^{i}$). In addition, the 3D_H_FMM had also generated a large arrival time error near the source point grid, as indicated in the red circle in Figure 6c. This was considered to have been mainly caused by the large wavefront curvature near the source point.

The eight receiving points R1 to R8, which are sensor coordinates, were next taken as examples in this study, and the theoretical values of the travel times were calculated according to Equation (6). The absolute error of travel time calculated by the 3D_H_FMM was calculated according to Equation (7), and then compared with those of the 3D_FMM. The comparison results are shown in Table 3. In this study, an absolute error lower than 1 × 10^−6^ ms was considered to be 0. According to the data presented in the table, the MAE of the travel time calculated by the 3D_FMM at the positions of the eight receiving points was 0.423 ms. Meanwhile, the MAE of the 3D_H_FMM was determined to be 0.055 ms, which was a reduction of 86.998% ($\left(\delta t_{\mathrm{abs},2}{}^{i}-\delta t_{abs,1}{}^{i}\right)/\delta t_{abs,1}{}^{i}$). Therefore, based on the results, it was clearly shown that for 3D microseismic velocity models, the method presented in this study had displayed the ability to calculate travel times more accurately at the receiving points when compared to the other examined method.

The main diagonal of the velocity model, in the direction of the line from the top left corner to the bottom right corner, was taken as the next example and the source point profile (three profiles where the source was located), and the entire velocity model (i.e., all nodes within the model) were analyzed in detail. The MAEs of multiple nodes calculated by the 3D_H_FMM at the travel time were calculated and compared with those of the 3D_FMM according to Equation (9). The comparison results are shown in Table 4. In terms of the MAE, the method presented in this study was lower than that of the 3D_FMM, and the overall MAE had been reduced by 88.335%. The source point profile was reduced by 73.377%, and the main diagonal of the velocity model was reduced by 76.572%. Therefore, the validity and veracity of the developed method were confirmed.

**Figure 7:**
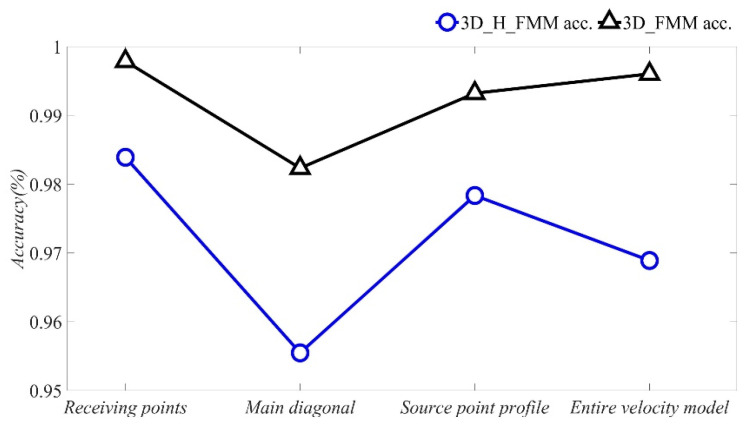
The accuracy of P-wave travel time calculated by 3D_H_FMM and 3D_FMM at multiple nodes in the Case A: small-scaled homogeneous model.

In the microseismic 3D velocity models, the accuracy of the overall travel time is mainly dependent on the diagonal 45° direction. In the current study, in order to verify that dependency, the accuracy of the travel times calculated using the 3D_H_FMM at multiple nodes was calculated according to Equation (10). The results are detailed in Figure 7. As can be seen from the figure, when compared with the 3D_FMM, the accuracy of the developed method was improved in multiple nodes, and the overall accuracy had been increased by 2.713%. Among those, the accuracy at the receiver points R1 to R4 was improved by 1.396%; the accuracy of the profile at the source point was improved by 1.486%, and the accuracy of the main diagonal direction of the velocity model was improved by 2.685%. Therefore, based on those findings, it could be concluded that the accuracy of the travel times of the entire velocity model had been mainly affected by the direction of the main diagonal at 45°. Furthermore, in the entire velocity model, when compared with the 3D_FMM, it was determined that the 3D_H_FMM was able to make up for the defects of larger errors in the travel times at the diagonal direction, thereby improving the extraction accuracy of the travel times in the entire space.

**Figure 8:**
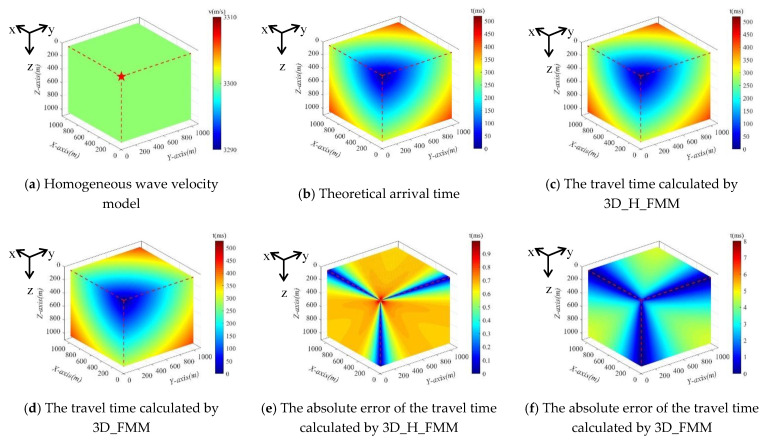
The Case B: large-scaled homogeneous model and its theoretical arrival time, the P-wave travel time, and error distribution map calculated by 3D_H_FMM and 3D_FMM.

Following the above-mentioned, Case B: large-scaled homogeneous model was set as 1000 m × 1000 m × 1000 m, and the sensor coordinate position is shown in Table 1. All corresponding experimental conditions are the same as Case A: small-scaled homogeneous model. Case B: large-scaled homogeneous model was shown in Figure 8a and the theoretical travel time was shown in Figure 8b. Both 3D_H_FMM and 3D_FMM were used to calculate the numerical solution of the P-wave travel time, respectively; see Figure 8c,d. The errors, namely the absolute errors, between the first phase travel time values determined by the 3D_H_FMM, 3D_ FMM, and its theoretical value were charted, as detailed in Figure 8e,f, respectively.

In the entire velocity model, the maximum theoretical value of the travel time was 519.615 ms, and the maximum first arrival travel times picked up, calculated by the 3D_H_FMM and 3D_FMM were 520.155 ms and 527.627 ms, respectively. The maximum absolute errors of the 3D_H_FMM and 3D_FMM were determined to be 0.997 ms and 8.012 ms, respectively. Therefore, based on the results, the maximum absolute errors calculated by the method developed in this study were far less than those of the 3D_FMM, at approximately 87.556%. The results of Case B: large-scaled homogeneous model were consistent with Case A: small-scaled homogeneous model, which fully demonstrates the effectiveness and accuracy of the developed method in the range of different model sizes.

Similarly, with Case B: large-scaled homogeneous model, R1–R8 were used to calculate the theoretical value of travel time, and 3D_H_FMM is used to calculate the travel time and absolute error, which formed a comparison with 3D_FMM. The comparison results were shown in Table 5.

According to the data, the MAE of the travel time is taken to be 4.423 ms for 3D_FMM at 8 receiver positions. Meanwhile, the MAE of 3D_H_FMM is determined to be 0.679 ms, which is reduced by 84.648% ($\left(\delta t_{\mathrm{abs},2}{}^{i}-\delta t_{abs,1}{}^{i}\right)/\delta t_{abs,1}{}^{i}$). The results of Case B: large-scaled homogeneous model were consistent with Case A: small-scaled homogeneous model, which significantly demonstrates the higher accuracy of the developed method.

The main diagonal of the velocity model, the source point profile, and the entire velocity model were analyzed in detail. The MAEs of multiple nodes were calculated by the 3D_H_FMM compared with those of the 3D_FMM. The comparison results are shown in Table 6. In terms of the MAE, the method presented in this study was lower than that of the 3D_FMM, and the overall MAE had been reduced by 90.593%. The source point profile was reduced by 78.203%, and the main diagonal of the velocity model was reduced by 81.402%. Therefore, the validity and veracity of the developed method were confirmed in the range of different model sizes.

### 3.2. Inhomogeneous Layered Velocity Model

In practical engineering, microseismic velocity models are often inhomogeneous. Therefore, this study established a layered velocity model for the purpose of simulating inhomogeneous geological bodies in experimental field tests. The goals were to verify the applicability and effectiveness of the developed method by accurately calculating the P-wave travel time. First of all, a three-dimensional layered velocity model was established. The P-wave velocities were random values between 2000 and 4000 m/s, and the source point S was randomly generated and irregularly distributed in the velocity model. Case C: small-scaled inhomogeneous velocity model was adopted, and there was a total of $50^{3}=125,000$ nodes. The specific scale range and the positions of sensors are shown below in Table 1.

**Figure 9:**
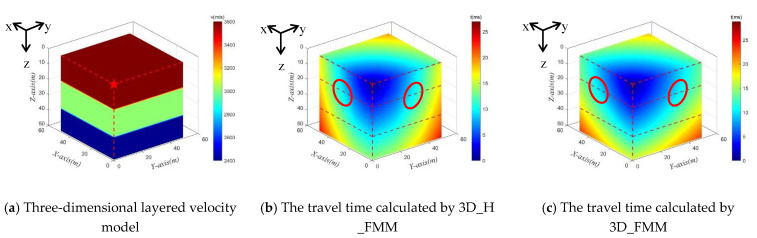
The Case C: small-scaled inhomogeneous velocity model and the travel time calculated by 3D_H_FMM and 3D_FMM.

Similar to Case A: small-scaled inhomogeneous velocity model, a single focal source was taken as an example for illustration purposes, as shown in Figure 9a. The velocities of the P-waves were 2400 m/s, 3000 m/s, and 3600 m/s. Then, assuming that the source point was at (1, 1, 1), both the 3D_H_FMM and the 3D_FMM were used to calculate the numerical solution of the travel time. The results are detailed in Figure 9b,c. The red dotted line in the figure corresponds to the layered interface, and the wavefront was located inside the red circle. In the entire velocity model, the maximum travel times calculated by the 3D_H_FMM and 3D_FMM were 26.973 ms and 28.808 ms, respectively. Those results also confirmed the applicability of the method developed in this study.

**Figure 10:**
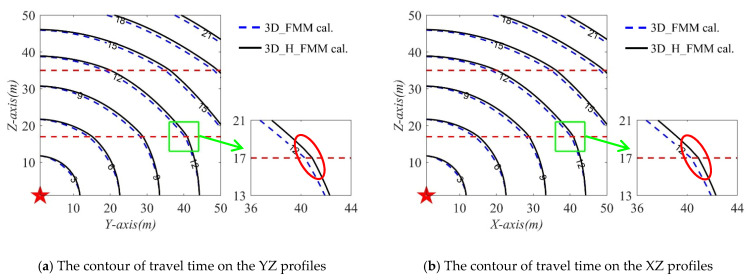

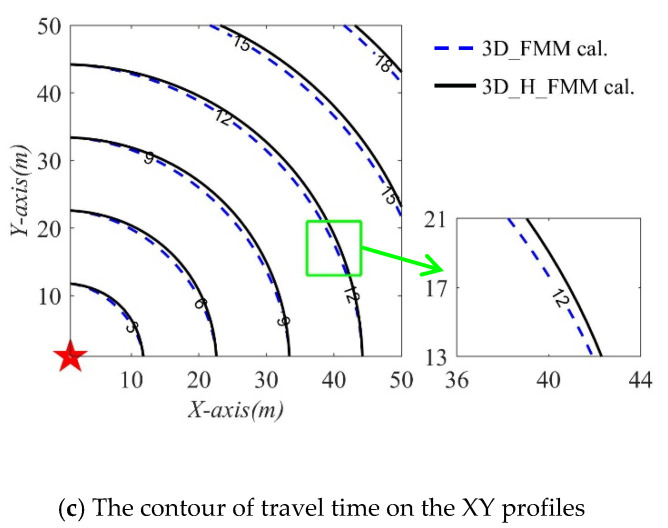
The contour of travel times of the three profiles of microseismic source point in the Case C: small-scaled inhomogeneous velocity model.

In order to verify the accuracy of the developed method in inhomogeneous layered velocity models, the travel time values of three sections (x = 1 m (YZ profile); y = 1 m (XZ profile), and z = 1 m (XY profile)) where the source point was located were calculated. The contour lines of the travel times determined by the 3D_H_FMM and 3D_FMM are shown in Figure 10. The red dotted line in the figure represents the corresponding layered interface, and the green frame line is the amplification of a small area of the layered interface. It can be seen in Figure 9 and Figure 10 that the trajectory of the numerical solution of the travel times calculated by the 3D_H_FMM and 3D_FMM in the layered velocity model were different in geometric form from those of the uniform model. For example, the wavefront shape changed significantly at the velocity interface, as shown in the red circle in the figure. Moreover, as can be seen from the contour distribution shown in Figure 10, the travel time of the 3D_H_FMM at a specific node was very close to that of the 3D_FMM. However, the 3D_H_FMM was slightly smaller. The verification results showed that the accuracy of the developed method was also improved when compared with the 3D_FMM under the conditions of an inhomogeneous velocity model, which verified that the developed method had a certain application potential for actual engineering data.

Following the above-mentioned, the Case D: large-scaled inhomogeneous velocity model was set as 1000 m × 1000 m × 1000 m, and the sensor coordinate position is shown in Table 1. All corresponding experimental conditions are the same as Case C: small-scaled inhomogeneous velocity model.

**Figure 11:**
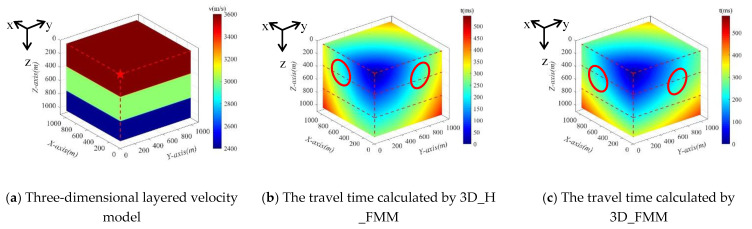
The Case D: large-scaled inhomogeneous velocity model and the travel time calculated by 3D_H_FMM and 3D_FMM.

Case D: large-scaled inhomogeneous velocity model was shown in Figure 11a. Both 3D_H_FMM and 3D_FMM were used to calculate the numerical solution of the P-wave travel time, respectively, see Figure 11b,c. The red dotted line in the figure corresponds to the layered interface, and the wavefront was located inside the red circle. In the entire velocity model, the maximum first arrival travel times picked up, calculated by the 3D_H_FMM and 3D_FMM were 544.479 ms and 576.199 ms, respectively. The results of Case D: large-scaled inhomogeneous velocity model were consistent with Case C: small-scaled inhomogeneous velocity model, which fully demonstrates the applicability of the method developed in this study in the range of different model sizes.

## 4. Validation of the 3D Microseismic Source Localization

Microseismic source localization was carried out using the travel times calculated by the developed method in order to verify the applicability and accuracy of the 3D_H_FMM in the complex rock mass wave velocity models. The model adopted the inhomogeneous three-layer layered medium model described in Section 2.2. The microseismic source point S was randomly generated and irregularly distributed in the velocity model at a scale range of 50 m^3^. The receiver positions R1 to R4 were consistent with the previous settings. The 3D_H_FMM method was used to calculate the grid nodes in the entire model in order to obtain the travel time values of the geophone receiver point. Then, a particle swarm optimization algorithm was used to locate the microseismic source point. The results were compared with those obtained using the 3D_FMM.

### 4.1. Localization Method Based on PSO Algorithms

A particle swarm optimization (PSO) algorithm is a type of nonlinear intelligent optimization algorithm with the ability to find optimal solutions through cooperation and information sharing among individuals within a group [39]. A PSO randomly selects multiple particles as the initial solution in a search space, and then determines the optimal solution through iteration. During each iteration, the particle keeps updating by tracking two “extremums” (for example, the population extremum and the individual extremum). The particle updates its velocity and position using the following formula:

(11) $$v_{i+1}=w\times v_{i}+c_{1}\times rand()\times (pbest_{i}-x_{i})+c_{2}\times rand()\times (gbest_{i}-x_{i})$$

(12) $$x_{i+1}=x_{i}+step\times v_{i}$$

where i represents the number of current iterations; *x_i_* and *v_i_* denote the current attributes of the particle: position and velocity; rand() is the uniformly distributed random number generated between [0, 1]; w indicates the inertia weight, whose size determines the global search ability of the particles and reflects the influence of individual historical achievements on existing achievements; $c_{1}$ and $c_{2}$ represent the self-learning ability and overall learning ability of particles, respectively. Setting *c*_1_ = *c*_2_ = 1, and where both are not 0, it is easier to maintain the balance between the convergence speed and the search effect, which is a better choice; $x_{i+1}$ and *v_i_*_+1_ are the updated position and speed, respectively, and *pbest_i_*, $gbest_{i}$ indicate the global best and personal best after the *i*th iteration, respectively.

**Figure 12:**
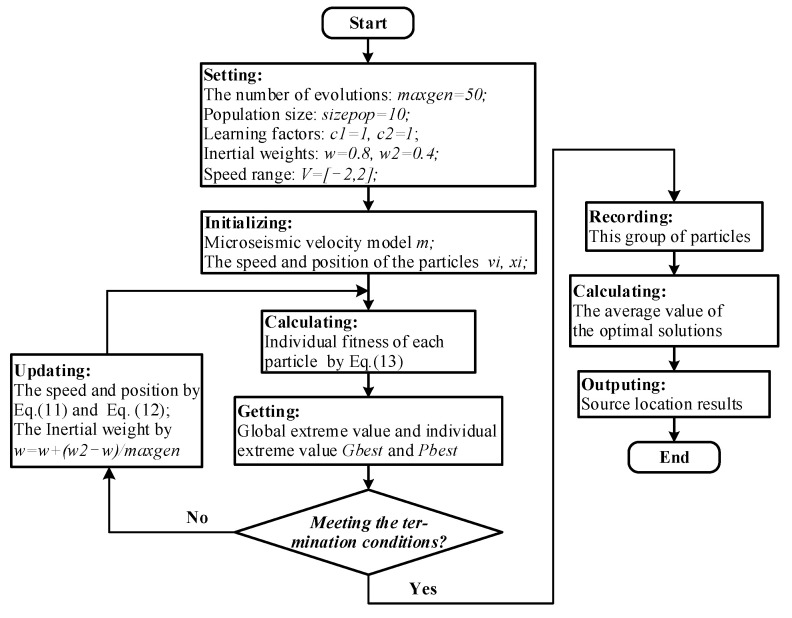
Process flow chart of microseismic source search based on PSO.

In this study, the microseismic source-target search space based on particle swarm optimization algorithm was three-dimensional, and 20 particles were set to form a community. The position of the ith particle was a three-dimensional vector, namely $x_{i}=(x_{i1},x_{i2},x_{i3})$, which represented the three-dimensional coordinate values in Cartesian coordinates. In addition, the velocity of the ith particle was also a three-dimensional vector, $v_{i}=(v_{i1},v_{i2},v_{i3})$. The optimal solution of the iterative search was the localization of the source point, and the objective function was as follows:

(13) $$f_{\min}=\sum_{i=1}^{N}\left|t_{A,obs}{}^{i}-t_{A,real}{}^{i}\right|$$

where i represents the *i*-th receiver; N indicates the number of receivers; $t_{A,obs}{}^{i}$ is the observed travel time of the assumed source point; $t_{A,real}{}^{i}$ denotes the actual travel time of the real source point, and its travel time value was calculated by both the 3D_H_FMM and the 3D_FMM. The process of microseismic source search based on PSO is detailed in Figure 12.

### 4.2. Localization Contrast Test and Result Analysis

For the purpose of showing the stability and robustness of the developed method, Case C and Case D are used to locating the microseismic source, respectively. Multiple particles were randomly selected as the assumed source points in a 3D space velocity model. Two positions on the layered interface, two positions at the bottom of the model, and two random positions were selected, with a total of six positions set as the source points. The 3D_H_FMM was used to calculate the travel times, and the travel times of each receiving point were recorded. Subsequently, a particle swarm optimization algorithm was used to locate and make a comparison with the 3D_FMM results.

**Figure 13:**
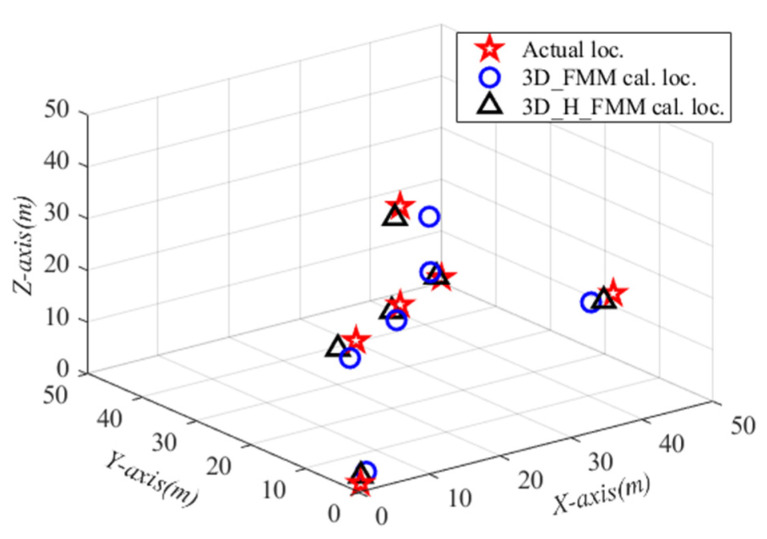
The localization results of microseismic sources with Case C: small-scaled inhomogeneous velocity model.

For each source point, use the travel time calculated by the eight receivers on the upper surface of the model to realize microseismic source localization. With Case C: small-scaled inhomogeneous velocity model, the average value of multiple particles of particle swarm was taken as the final localization result of the microseismic source, as shown in Figure 13. As can be seen in the figure, 3D_H_FMM and 3D_FMM were able to achieve more accurate microseismic source localization. However, when compared with 3D_FMM, the localization results using 3D_H_FMM were found to be closer to the real source point.

**Figure 14:**
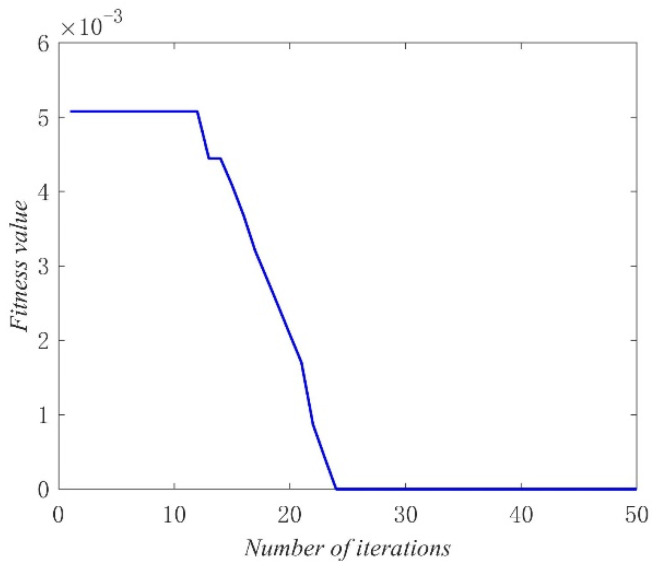
The example curve of the optimal individual fitness function.

The source point (1, 1, 1) was taken as an example in the process of microseismic source localization using particle swarm optimization algorithm in this study. The optimal individual fitness value function is shown in Figure 14. In the velocity model with a scale range of 50 m^3^, the maximum evolution coefficient was set as 50, and the population size was set as 10. The particle swarm optimization algorithm had the ability to converge after 24 iterations, and the adaptive value function of the optimal individual could directly identify the global optimal position point.

The localization results of multiple microseismic source points with Case C: small-scaled inhomogeneous velocity model are summarized in Table 7. The results showed that the error fluctuation of the source localization results calculated by 3D_H_FMM and 3D_FMM was small. The mean localization errors (Mean loc. err.) of the results at different source points were 1.901 m and 3.447 m, respectively, while the median of positioning errors (Medi. loc. err.) was 1.847 m and 3.459 m, for the two methods. This study found that when compared with 3D_FMM, the microseismic source localization errors of the developed method had been reduced by 1.546 m, and the localization accuracy of the three-dimensional space was significantly improved.

In addition, a 5% arrival error is added to the P-wave propagation time as Gaussian white noise in the synthetic event localization test. The mean and median absolute errors of the positioning results at different source points when traveling with noise-containing P-wave are 2.880 m and 2.796 m, respectively. Therefore, the results confirmed the applicability and accuracy of the solution of the travel times achieved by the 3D_H_FMM in inhomogeneous layered velocity models and further proved the robustness and superiority of the developed method. Positioning errors are inevitable when using the time difference method for microseismic source localization. We must consider the influence of the algorithm on the results.

**Figure 15:**
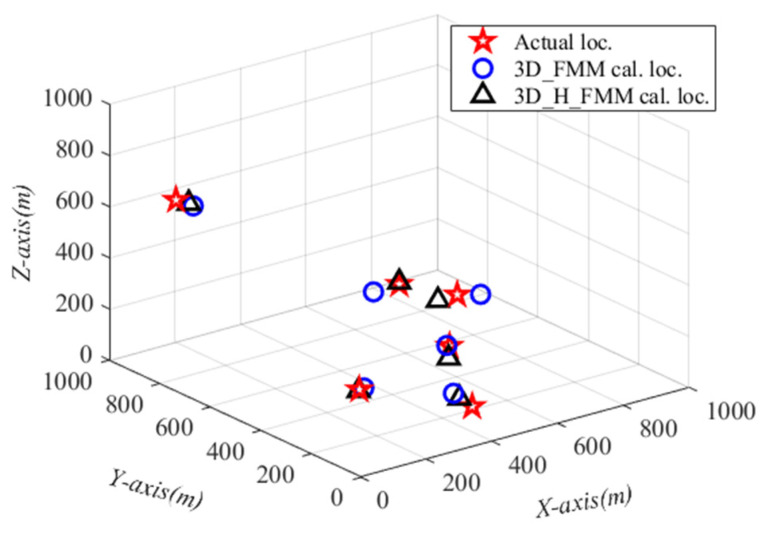
The localization results of microseismic sources with Case D: large-scaled inhomogeneous velocity model.

Similarly, with Case D: large-scaled inhomogeneous velocity model, selecting two positions near the center of the layered interface, two positions near the edge of the model, and two positions at the bottom of the model, setting a total of six positions as source points. The average value of multiple particles of particle swarm was taken as the final localization result of the microseismic source, as shown in Figure 15. The localization results were consistent with that of Case C: small-scaled inhomogeneous velocity model; the localization results using 3D_H_FMM were found to be closer to the real source point compared with 3D_FMM.

The localization results of multiple microseismic source points with Case D: large-scaled inhomogeneous velocity model are summarized in Table 8. Then, five percent of the Gaussian white noise was added to the P-wave propagation time in the synthetic event localization test.

The MAEs of the localization results at different source points were 42.764 m and 62.092 m, respectively, while the median of absolute errors was 41.379 m and 61.869 m, for the two methods. This study found that when compared with 3D_FMM, the microseismic source localization errors of the developed method had been reduced by 19.328 m, and the localization accuracy of the three-dimensional space was significantly improved. The mean and median absolute errors of the positioning results at different source points when traveling with noise-containing P-wave are 49.157 m and 45.677 m, respectively.

### 4.3. Discussion

The 3D_H_FMM has obvious advantages in computational accuracy. 3D_H_FMM mainly compensates for the large error of travel time in the diagonal direction, thus improving the accuracy in the whole space. The accuracy of 3D_H_FMM is improved the most in the diagonal direction. In the large-size or small-size microseismic experiments, the closer to the middle of the model (that is, the closer the direction of the propagation path from the source to the sensor is to the diagonal direction), the better the localization effect of 3D_H_FMM than 3D_FMM. The method in this paper is consistent in reducing the errors in the travel time calculation and the errors in the baseline commutation localization results.

Due to the limitations of the localization method itself, the localization results error is inevitable, as well as the influence of noise. In the positioning test, we added a 5% arrival time error to test the robustness of the method. By quoting the developed 3D_H_FMM method, this paper reduces the positioning error, which cannot be completely eliminated.

## 5. Conclusions

In this research investigation, a 3D high-order fast marching method was developed to calculate the travel times in microseismic 3D velocity models. The developed method was validated in both homogeneous velocity models and inhomogeneous layered media velocity models of different scales, respectively. On the basis of the results obtained in this study’s experiments, a particle swarm optimization algorithm was used to locate the microseismic source. The 3D_FMM was used as a comparison method in this study, and the main conclusions were as follows:

(1) The validity and accuracy of the developed method were verified in homogeneous velocity models. It was found that in a three-dimensional model, the overall mean absolute error (MAE) of the two homogenous models using 3D_H_FMM had been reduced by 88.335% with small-scaled homogeneous model, and 90.593% with the large-scaled homogeneous model, compared with the traditional 3D_FMM. The developed method made up for the large error in the diagonal direction, and improved the extraction accuracy of the full space travel time. Therefore, the validity and veracity of the developed method were fully proven.

(2) The applicability and robustness of the developed method were also verified in a model of inhomogeneous layered wave velocities. The method presented in this study had certain application potential for actual engineering data. The localization results revealed that the 3D_H_FMM was closer to the real source, Moreover, the Mean loc. err. Medi. loc. err. of the localization results at different source points in 3D space were 1.901 m, 1.847 m, while 3D_FMM’s were 3.447 m and 3.459 m with the small-scaled inhomogeneous model; the Mean loc. err., Medi. loc. err of the localization results at different source points in 3D space were 42.764 m and 41.379 m, while 3D_FMM’s were 62.092 m and 61.869 m with the large-scaled inhomogeneous model. Additionally, add to the arrival time noise to verify the robustness. Therefore, based on this study’s results, the superiority of the developed method was verified.

The method developed in this study provided certain technical support for the travel time computation and source localization of microseismic monitoring processes, and was considered to have good engineering application prospects. However, due to the complexity of inhomogeneous geological bodies in actual engineering projects, the application potential of this method for actual data will require further research in the field.

## Figures and Tables

**Table 1:** The cases of microseismic velocity models, including model scale size and sensor coordinates.

Case A and Case C			Case B and Case D		
Model Scale	Sensor Coordinates (m)		Model Scale	Sensor Coordinates (m)	
50 m × 50 m × 50 m	R1	(1, 1, 50)	1000 m × 1000 m × 1000 m	R1	(10, 10, 1000)
	R2	(1, 50, 50)		R2	(10, 1000, 1000)
	R3	(50, 1, 50)		R3	(1000, 10, 1000)
	R4	(50, 50, 50)		R4	(1000, 1000, 1000)
	R5	(10, 20, 50)		R5	(100, 200, 1000)
	R6	(10, 40, 50)		R6	(100, 400, 1000)
	R7	(30, 20, 50)		R7	(300, 200, 1000)
	R8	(30, 40, 50)		R8	(300, 400, 1000)

**Table 2:** The main notations used throughout the paper.

Symbol	Definition
v	The range of P-wave velocity values
S	The source point in microseismic monitoring, abbreviated as S
R	The receiver points, which are sensors’ coordinates in microseismic monitoring, referred to as R, contains R1–R8
$t_{A}{}^{i}$	The analytical solution at the node i, which is theoretical value of P-wave travel time
$t^{i}$	The numerical solution at the node i, which is calculated by 3D_H_FMM and 3D_FMM
max.t	Maximum value of P-wave travel time
$\delta t_{\mathrm{abs}}{}^{i}$	The absolute error at the node i between the analytical and numerical solution
$\upsilon t_{acc}{}^{i}$	Degree of accuracy of P-wave travel time
*max.err.*	Maximum value of absolute error
MAE	Mean absolute error
MAE red. per.	$\left(\delta t_{\mathrm{abs},2}{}^{i}-\delta t_{abs,1}{}^{i}\right)/\delta t_{abs,1}{}^{i}$, the percentage reduction in MAE when traveling with the developed method 3D_H_FMM, compared with 3D_FMM
Mean loc. err.	Mean error in microseismic source localization
Medi. loc. err.	Median error in microseismic source localization

**Table 3:** The travel time and its absolute error at the receiving points of R1 to R8 calculated by 3D_H_FMM and 3D_FMM in the Case A: small-scaled homogeneous model.

Receiving Points (m)	Theoretical (ms)	3D_FMM Cal. (ms)	3D_FMM Abs. Err. (ms)	3D_H_FMM Cal. (ms)	3D_H_FMM Abs. Err. (ms)
R1 (1, 1, 50)	14.848	14.848	0	14.848	0
R2 (50, 1, 50)	20.999	21.395	0.396	21.066	0.067
R3 (50, 1, 50)	20.999	21.395	0.396	21.066	0.067
R4 (50, 50, 50)	25.718	26.403	0.685	25.773	0.055
R5 (10, 20, 50)	16.158	16.490	0.332	16.223	0.065
R6 (10, 40, 50)	19.173	19.629	0.456	19.240	0.067
R7 (30, 20, 50)	18.189	18.699	0.510	18.247	0.058
R8 (30, 40, 50)	20.914	21.525	0.611	20.975	0.061
MAE (ms)	–	–	0.423	–	0.055

**Table 4:** The MAE of the travel time calculated by 3D_H_FMM and 3D_FMM at multiple nodes in the Case A: small-scaled homogeneous model.

Multiple Nodes	3D_FMM Abs. Err. (ms)	3D_H_FMM Abs. Err. (ms)	MAE Red. Per. (%) $\left(\delta t_{\mathrm{abs},2}{}^{i}-\delta t_{abs,1}{}^{i}\right)/\delta t_{abs,1}{}^{i}$
Source point profile	0.220	0.059	73.377
Main diagonal	0.304	0.071	76.572
Entire velocity model	0.422	0.049	88.335

**Table 5:** The travel time and its absolute error at the receiving points of R1 to R8 calculated by 3D_H_FMM and 3D_FMM in the Case B: large-scaled homogeneous model.

Receiving Points (m)	Theoretical (ms)	3D_FMM Cal. (ms)	3D_FMM Abs. Err. (ms)	3D_H_FMM Cal. (ms)	3D_H_FMM Abs. Err. (ms)
R1 (10, 10, 1000)	300	300	0	300	0
R2 (1000, 10, 1000)	424.264	424.925	0.661	424.925	0.661
R3 (1000, 10, 1000)	424.264	428.936	4.672	424.925	0.661
R4 (1000, 1000, 1000)	519.615	527.627	8.012	520.155	0.540
R5 (100, 200, 1000)	306.690	308.587	1.897	307.161	0.471
R6 (100, 400, 1000)	323.590	326.906	3.316	324.244	0.653
R7 (300, 200, 1000)	317.864	321.211	3.346	318.318	0.454
R8 (300, 400, 1000)	334.200	338.832	4.632	334.833	0.633
MAE (ms)	–	–	4.423	–	0.679

**Table 6:** The MAE of the travel time calculated by 3D_H_FMM and 3D_FMM at multiple nodes in the Case B: large-scaled homogeneous model.

Multiple Nodes	3D_FMM Abs. Err. (ms)	3D_H_FMM Abs. Err. (ms)	MAE Red. Per. (%) $\left(\delta t_{\mathrm{abs},2}{}^{i}-\delta t_{abs,1}{}^{i}\right)/\delta t_{abs,1}{}^{i}$
Source point profile	2.654	0.578	78.203
Main diagonal	3.703	0.689	81.402
Entire velocity model	5.026	0.473	90.593

**Table 7:** The localization results of microseismic sources using travel times (calculated by 3D_H_FMM and 3D_FMM) and their noise-laden travel times with Case C: small-scaled inhomogeneous velocity model.

Number	Actual Point S (m)	Velocity Model (m/s)	3D_H_FMM Loca. Results (m)	3D_H_FMM Abs. Err. (m)	3D_FMM Loca. Results (m)	3D_H_FMM Abs. Err. (m)	3D_H_FMM Loca. Results with Time Noise (m)	3D_H_FMM Abs. Err. with Time Noise (m)
1	(1, 1, 1)	(2100, 3100, 3600)	(1.893, 2.029, 1.608)	1.492	(3.479, 3.210, 1.107)	3.323	(1.935, 2.746, 2.512)	2.455
2	(50, 50, 1)	(2300, 2700, 3800)	(48.532, 49.084, 2.169)	2.088	(47.431, 48.620, 3.488)	3.833	(48.241, 48.286, 2.579)	2.919
3	(25, 25, 16)	(2500, 3100, 3900)	(24.394, 25.883, 14.786)	1.619	(23.035, 23.264, 14.576)	2.984	(22.145, 23.896, 14.643)	3.348
4	(25, 25, 35)	(2000, 3300, 4000)	(23.691, 24.158, 33.636)	2.070	(27.841, 23.539, 32.753)	3.906	(28.127, 23.925, 35.414)	3.332
5	(18, 24, 12)	(2600, 3000, 3400)	(16.397, 25.291, 10.551)	2.517	(16.107, 22.764, 9.959)	3.046	(15.815, 25.350, 12.739)	2.673
6	(42, 8, 20)	(2400, 2800, 3600)	(40.560, 7.631, 19.348)	1.623	(40.556, 10.390, 17.738)	3.594	(42.418, 9.651, 18.257)	2.552
Mean loc. err. (m)	–	–	–	1.901	–	3.447	–	2.880
Medi. loc. err. (m)	–	–	–	1.847	–	3.459	–	2.796

**Table 8:** The localization results of microseismic sources using travel times (calculated by 3D_H_FMM and 3D_FMM) and their noise-laden travel times with Case D: large-scaled inhomogeneous velocity model.

Number	Actual Point S (m)	Velocity Model (m/s)	3D_H_FMM Loca. Results (m)	3D_H_FMM Abs. Err. (m)	3D_FMM Loca. Results (m)	3D_H_FMM Abs. Err. (m)	3D_H_FMM Loca. Results with Time Noise (m)	3D_H_FMM Abs. Err. with Time Noise (m)
1	(500, 500, 350)	(2500, 3200, 3600)	(477.127, 470.127, 379.872)	48.041	(453.344, 541.405, 317.331)	70.416	(456.977, 469.634, 378.505)	59.880
2	(600, 400, 320)	(2100, 2400, 3800)	(564.570, 430.354, 296.373)	52.296	(644.396, 364.939, 321.473)	56.590	(579.493, 426.349, 290.993)	44.229
3	(10, 20, 330)	(2800, 3000, 3900)	(28.565, 46.882, 307.532)	39.650	(50.342, 54.385, 307.187)	57.708	(41.486, 26.192, 300.854)	43.350
4	(50, 800, 700)	(2000, 3500, 4000)	(75.643, 782.911, 686.016)	33.840	(81.652, 772.851, 677.864)	47.212	(47.859, 763.772, 677.478)	42.712
5	(500, 300, 200)	(2600, 3000, 3400)	(482.247, 280.575, 168.159)	41.308	(460.710, 259.671, 234.494)	66.030	(475.462, 275.235, 168.293)	47.125
6	(400, 80, 100)	(2300, 2700, 3500)	(382.262, 107.249, 125.709)	41.450	(356.784, 97.921, 158.102)	74.597	(357.357, 118.416, 105.388)	57.647
Aver. err. (m)	–	–	–	42.764	–	62.092	–	49.157
Medi. err. (m)	–	–	–	41.379	–	61.869	–	45.677

## Data Availability

Not applicable.
